# Information-Theoretical Analysis of EEG Microstate Sequences in Python

**DOI:** 10.3389/fninf.2018.00030

**Published:** 2018-06-01

**Authors:** Frederic von Wegner, Helmut Laufs

**Affiliations:** ^1^Epilepsy Center Rhein-Main, Goethe University Frankfurt, Frankfurt am Main, Germany; ^2^Department of Neurology and Brain Imaging Center, Goethe University Frankfurt, Frankfurt am Main, Germany; ^3^Department of Neurology, University Hospital Kiel, Kiel, Germany

**Keywords:** EEG microstates, information theory, entropy, mutual information, Markovianity, open-source

## Abstract

We present an open-source Python package to compute information-theoretical quantities for electroencephalographic data. Electroencephalography (EEG) measures the electrical potential generated by the cerebral cortex and the set of spatial patterns projected by the brain's electrical potential on the scalp surface can be clustered into a set of representative maps called EEG microstates. Microstate time series are obtained by competitively fitting the microstate maps back into the EEG data set, i.e., by substituting the EEG data at a given time with the label of the microstate that has the highest similarity with the actual EEG topography. As microstate sequences consist of non-metric random variables, e.g., the letters A–D, we recently introduced information-theoretical measures to quantify these time series. In wakeful resting state EEG recordings, we found new characteristics of microstate sequences such as periodicities related to EEG frequency bands. The algorithms used are here provided as an open-source package and their use is explained in a tutorial style. The package is self-contained and the programming style is procedural, focusing on code intelligibility and easy portability. Using a sample EEG file, we demonstrate how to perform EEG microstate segmentation using the modified K-means approach, and how to compute and visualize the recently introduced information-theoretical tests and quantities. The time-lagged mutual information function is derived as a discrete symbolic alternative to the autocorrelation function for metric time series and confidence intervals are computed from Markov chain surrogate data. The software package provides an open-source extension to the existing implementations of the microstate transform and is specifically designed to analyze resting state EEG recordings.

## 1. Introduction and background

Electroencephalography (EEG) is a routine technique in neuroscientific research and the clinical sciences, used to measure electrical potentials generated by the cerebral cortex. It is a relatively low-cost and widely distributed diagnostic tool. The measured EEG signal records a superposition of excitatory and inhibitory postsynaptic potentials via electrodes located on the skull surface (Niedermeyer and da Silva, 2005). Among the data reduction techniques that have been employed to compress EEG recordings, the microstate algorithm is of special importance as it has been evaluated in a variety of experimental conditions (Lehmann et al., 1987; Wackermann et al., 1993; Pascual-Marqui et al., 1995). The microstate algorithm can be summarized as follows. Consider an EEG data set that consists of n_t_ time samples from n_ch_ channels, or electrode locations. Then, each sample is an array of n_ch_ real numbers, each number representing the electrical potential at a specific location, and the whole array provides a discrete sampling of the continuous electrical field. An EEG data set can therefore be visualized as a time series of changing spatial patterns, often called maps. The microstate algorithm searches for a small set of spatial patterns that explain the maximum amount of the data's variance. Often, only four representative maps are needed to explain ca. 70% of the data variance and to capture neurobiologically relevant data features (Koenig et al., 2002; Brodbeck et al., 2012; Khanna et al., 2015; Kuhn et al., 2015). It could be shown that the computed microstates convey information about functional brain states during cognition (Milz et al., 2015), different vigilance states (Brodbeck et al., 2012; Kuhn et al., 2015), and disease (Koenig et al., 1999; Nishida et al., 2013). We here implement the commonly employed modified K-means algorithm, as introduced in Pascual-Marqui et al. (1995) and which has been used in many published studies, for instance in vision research (Antonova et al., 2015), studies on olfaction (Iannilli et al., 2013), and taste (Iannilli et al., 2017), and in a multi-center schizophrenia study (Lehmann et al., 2005), to name but a few.

The canonical K-means algorithm yields a cluster assignment that minimizes the sum of squared distances of all data points to their respective cluster centroids, i.e., to the arithmetic mean of all points currently assigned to that cluster. The algorithm proceeds stochastically, using a fixed number of clusters and initializing the cluster centroids with randomly selected data samples. In the case of EEG records, a data sample consists of an array of electrical potential values at a given time point, and the size of the array represents the number of EEG channels. In each iteration, the algorithm assigns each data sample to its closest cluster centroid, and then updates all clusters and their centroids taking into account the newly assigned samples. Modified K-means clustering for EEG microstates, as introduced in Pascual-Marqui et al. (1995), does not use the arithmetic mean of samples to represent the cluster, but the first principal component of the samples. Thus, the polarity of the EEG topography is ignored, leaving the overall symmetry of the potential topography as the feature to be clustered (Wackermann et al., 1993; Pascual-Marqui et al., 1995). The convergence criterion for the modified K-means algorithm is the relative error in the explained variance, as detailed further below.

In this context, two particular types of EEG experimental designs should be mentioned, resting state recordings on the one hand, and event-related potentials (ERP) on the other. We here focus on resting state recordings, in which the ongoing EEG in a task-free (“resting”) condition is recorded in order to follow spontaneous changes in cortical activity. Resting state recordings have received considerable attention as they provide insight into functional brain networks that spontaneously activate and de-activate (Tagliazucchi et al., 2012). In ERP experiments, which we will not study further in this article, certain stimuli (acoustic, visual, cognitive tasks) are presented repetitively and the synchronously recorded EEG signal is analyzed in blocks. The start of each EEG data block is aligned with the stimulus presentation times, and thus, EEG features time-locked to stimulus onset are extracted. Due to often low signal-to-noise ratios in single stimulus responses, the evoked EEG changes are usually averaged. We mention ERP experiments since the microstate approach has been applied to ERP data for a long time (Murray et al., 2008). The algorithms presented here can readily be applied to ERP data sets, however, we do not provide the functionalities for the necessary pre-processing, such as epoch splitting, averaging, and ERP component identification.

The microstate algorithm transforms an EEG data set into a sequence of microstate labels, according to maximum similarity between the candidate microstates and the actual EEG topography. Commonly, the microstate maps are labeled with the symbols A–D. The fact that the resulting time series consist of categorical variables severely limits the list of applicable time series methods. Frequently used linear characteristics, such as the autocorrelation function or the power spectral density for instance, cannot be computed as sum and product terms are not defined on the discrete set of states.

The most frequently used approach is the transition matrix method that summarizes microstate dynamics by a square matrix of first-order transition statistics, i.e., the conditional probabilities of transitioning from one microstate to the next (Wackermann et al., 1993). The main limitation of this approach is the fact that only the *t* → *t* + 1 transition, i.e., a single time lag is considered. On a conceptual level, the transition matrix can only fully represent a first-order Markov process, for which the complete information about the future state *X*_*t*+1_ is contained in the random variable *X*_*t*_. We have shown that resting state EEG microstate sequences do not follow the Markov property, when testing Markovianity of the time series statistically (von Wegner et al., 2017). EEG microstate sequences rather show memory effects extending up to time scales of several hundred milliseconds (von Wegner et al., 2017). The statistical tests for Markovianity of order 0, 1, and 2 are contained in the software package introduced here.

As an alternative, a random walk analysis of microstate sequences has been proposed (Van de Ville et al., 2010). To use the method, however, the microstate labels (e.g., A–D) have to be mapped to real numbers, e.g., ±1, in order to use Hurst exponent estimators. Furthermore, the method aggregates several microstates into one class which is mapped to a single real number (Van de Ville et al., 2010; von Wegner et al., 2016). In the case of four microstates, an arbitrary pair of microstates is mapped to the value −1, and the other two microstates are mapped to the value +1. This procedure has several disadvantages. First, there are no biologically inspired reasons which microstate maps should be grouped into one class. If all possible class assignments are tested independently, their number diverges exponentially for larger numbers of microstates. In the case of an odd number of microstates, the partition into two classes is even more difficult to justify. Second, the arithmetic operations performed on the assigned real numbers (sums, products, square roots) do not have a clearly defined meaning on the level of the EEG potential topographies they represent.

Finally, the transition matrix approach and the random walk embedding contradict each other on a theoretical level, as the first uses a memory-less Markov model and the latter uses an infinite memory, scale-free approach. To overcome these limitations, in a recent publication we introduced information-theoretical methods in order to (i) work with an arbitrary number of microstate labels directly, and (ii) to assess the memory structure of microstate sequences for all time lags, i.e., beyond *t* → *t*+1 transitions, as captured by the transition matrix method (von Wegner et al., 2017). We also added further statistical tests for stationarity and symmetry of the transition matrix, and finally detected previously unrecognized periodicities in microstate sequences by means of the time-lagged mutual information function. Our previous publication provides an evaluation of these methods on a set of healthy subject resting state EEG recordings. In the present work, we make the methods developed and analyzed in von Wegner et al. (2017) available to other researchers. The code provided along with this manuscript allows to reproduce our previous results, and to perform new studies using the same methodology.

## 2. Software design

The philosophy of this project is to provide a free and open-source stand-alone package. We chose to implement the algorithm in Python (Rossum, 1995) in order to provide a freely distributable, open-source, cross-platform implementation without restrictions with respect to licensed or commercial software, or the operating system used. Moreover, Python offers easily accessible source code and a reasonable trade-off between performance and code comprehensibility.

The programming style is procedural, providing a set of functions to import, pre-process, visualize, and analyze EEG data sets with the microstate algorithm and the information-theoretical metrics described in von Wegner et al. (2017). Individual functions can easily be imported into other Python projects, and the procedural approach facilitates portability, compared to an object-oriented approach introducing specific class structures for EEG data that may interfere with data structures defined in other software packages.

From our experience, the scientific process often starts with a visual exploration phase, followed by more extensive and often automated data analyses, where the code may run on headless servers or remote computation facilities using the command line. Therefore, we have chosen to implement the software as a command line tool, to be used in scripts aimed at high productivity, i.e., to process a hierarchy of directories containing the EEG files to be analyzed. Rather than providing a graphical user interface (GUI), we use an interactive IPython notebook tutorial for visual analysis during the data exploration phase. The notebook format can easily be extended and modified. By this means, preliminary and intermediate results can directly be used in a tutorial or presentation setting. Finally, as another argument to use Python and as a perspective into the processing of larger EEG databases, Python code allows straightforward extensions to database management and web applications while staying within the same programming framework. The code is provided as a single source code file in Python 2.7 syntax and using standard code documentation following the PEP 8 style guide.

### 2.1. Requirements and dependencies

We provide stand-alone code using minimal dependencies. Dependencies consist in standard Python packages for scientific computing and visualization and have no link to licensed or commercial software. In order to run the tutorials provided in the source code and in the IPython notebook, the user needs to have installed:

- NumPy for array handling and numerical computing.
- matplotlib for data visualization.
- SciPy for filtering, interpolation, and χ^2^ statistics.
- StatsModels for multiple comparison statistics.
- scikit-learn for principial component analysis based EEG data visualization.

When only using the core functions implementing the microstate algorithm, statistical tests and information-theoretic quantities, the packages StatsModels (for multiple comparisons) and scikit-learn (for principal component analysis based visualization) can be omitted.

### 2.2. Command line options

From the command line, the following options are available:

- “-i” or “- -input” defines the full path to a single “.edf” file to be processed.
- “-f” or “- -filelist” defines the full path to a text file containing the full paths to all “.edf” files to be processed, row-wise.
- “-d” or “- -directory” defines the path to a directory, from which all “.edf” files will be processed.
- “-m” or “- -markovsurrogates” sets the number of Markov surrogates to be used for the computation of the mutual information confidence interval.

On the command line, all options can be viewed running the source file with the –help option.

### 2.3. Other implementations of the microstate algorithm

A detailed description of the original microstate algorithm is given in Pascual-Marqui et al. (1995) and Murray et al. (2008). Computational implementations exist as the Windows executable Cartool which is described in Brunet et al. (2011). The program is freely available, but ships without source code. For the commercial Matlab software, a free and open-source implementation called microstates has been published, and the package depends on the Matlab EEGLAB toolbox. Another EEGLAB-based implementation is the Microstate-EEGlab-toolbox. The large Chicago Electrical Neuroimaging Analytics software package contains the microstate algorithm as a Matlab plugin for the Brainstorm software. Recently, the probabilistic microstate analysis approach was published (Dinov and Leech, 2017), where microstates were obtained by the classical K-means algorithm rather than the modified K-means algorithm as given in Murray et al. (2008). The Key institute Python implementation for ERP analysis contains the microstate algorithm as presented in Milz (2016).

We would like to highlight that none of the implementations listed above includes the information-theoretical analyses contained in our package. However, as the other packages contain alternative implementations of the microstate algorithm and further analytic approaches, the user may choose to use and benefit from interfacing our code with some of these software packages. This is especially easy in the case of communication with external Python code. Our code allows for easy portability to other Python programs by simply importing the corresponding functions, e.g., the Markov tests, entropy calculations, surrogate data synthesis, and the implementation of the mutual information function.

## 3. The processing pipeline

**Figure 1:**
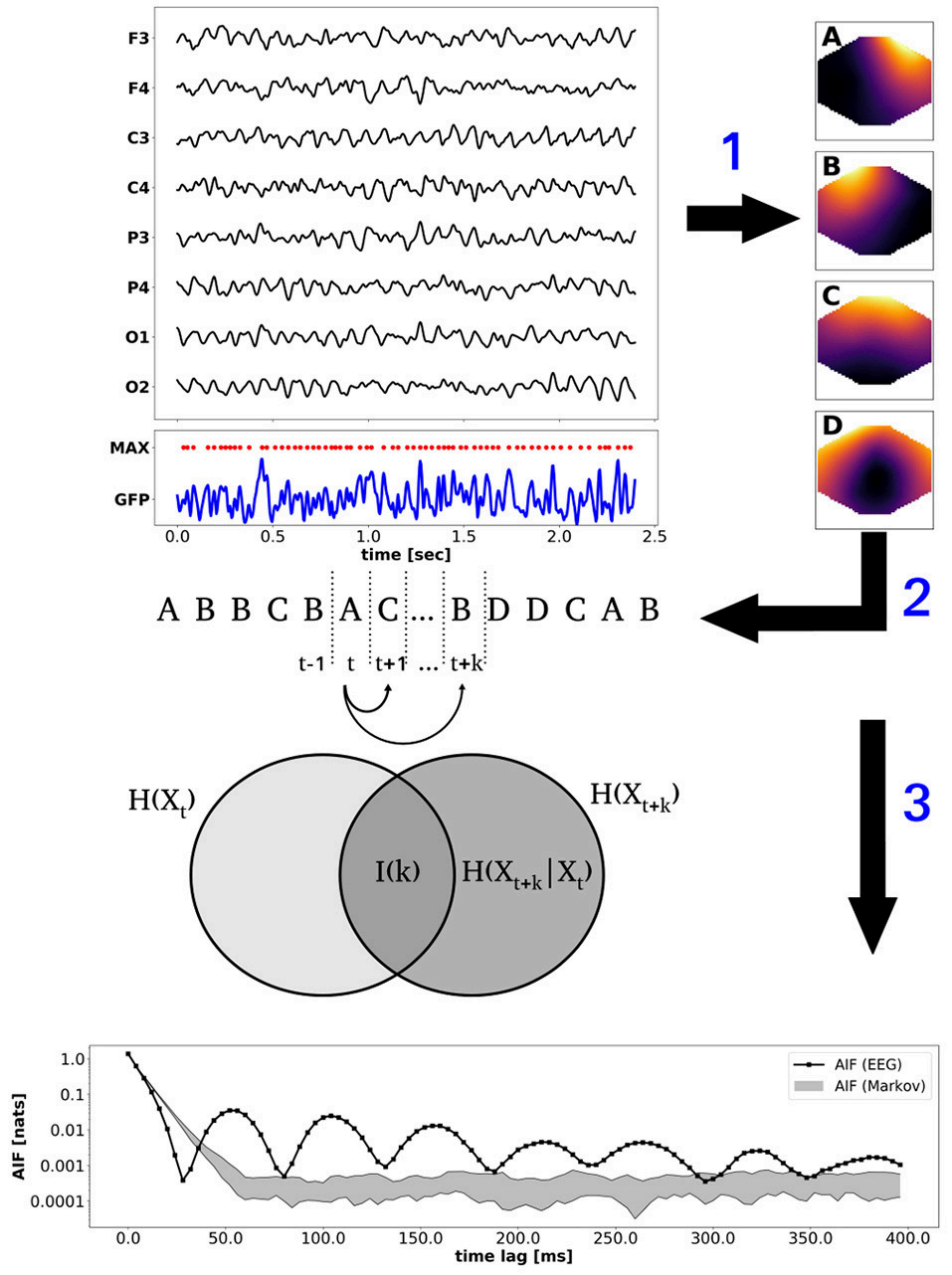
Algorithm: the **(top)** panel shows a section of resting state EEG (1–40 Hz, black), the global field power (GFP, blue), and the local GFP maxima (red dots). EEG topographies at local GFP maxima are clustered by the modified K-means algorithm to obtain the *n* = 4 microstate maps A–D (step 1). Fitting the maps back into the EEG data set yields the microstate sequence (A,B,B,C,…) depicted below the EEG data (step 2). Step 3 illustrates information-theoretical analysis of the microstate sequence. Time-lagged mutual information *I*(*k*) for time lag *k* is illustrated by a Venn diagram. The **(bottom)** panel shows the periodic mutual information function (black) and the Markov confidence interval (gray area, α = 0.01).

In the following, we illustrate a typical processing pipeline that can be implemented with the provided functions. During presentation, it should become clear that not all presented computations have to be performed, or necessarily in the order presented in the example. In particular, the number of clusters to be computed is provided by the user, and any integer (≥ 2) is allowed. All subsequent analysis steps are not affected by the choice and are computed for the given number of clusters. The Figure 1 summarizes the procedure. Selected EEG channel data is illustrated on the top left, showing the channel abbreviations on the y-axis (e.g., O1 is the left occipital electrode). Below, the global field power (GFP, blue) and its local maxima (MAX, red dots) are shown. The EEG topographies at the local GFP maxima provide the input for modified K-means clustering (arrow 1). The clustering procedure yields the four microstate maps A–D shown on the right. Step 2 refers to the competitive back-fitting of the microstate maps into the EEG data set, based on a maximum squared correlation metric. The microstate time series is illustrated by the label sequence A, B, B, C…depicted below the EEG data. Step 3 corresponds to information-theoretical analysis, in particular to time-lagged mutual information. The Venn diagram visualizes mutual (or shared) information between the microstates at time point *t* and the k-step future time point *t* + *k* as the intersection between two sets representing the entropies *H*(*X*_*t*_) and *H*(*X*_*t*+*k*_). Equivalently, mutual information is defined as *I*(*k*) = *H*(*X*_*t*+*k*_) − *H*(*X*_*t*+*k*_ | *X*_*t*_), i.e., as the difference between the uncertainty about state *X*_*t*+*k*_, and the uncertainty about *X*_*t*+*k*_ given exact knowledge about *X*_*t*_. To put it differently, *I*(*k*) measures the information about *X*_*t*+*k*_ that is contained in *X*_*t*_. Step 3 points to the bottom panel of Figure 1, which shows the time-lagged mutual information function for time lags *k* up to 400 ms. To point out non-Markovian memory effects in experimental EEG data, we show the mutual information function for a microstate sequence from experimental EEG data, as well as a confidence interval computed from 10 Markov surrogate sequences (significance level α = 0.01). Time-lagged mutual information of EEG data is shown in black (solid line with black squares) and the Markov confidence interval is shown as a gray-shaded area. The experimental mutual information function shows distinct oscillatory peaks not explained by the Markov model. Further details are given in the subsequent sections.

### 3.1. EEG data and pre-processing

To run this tutorial, we provide a test EEG file (test.edf) which must be located in the same folder as the source code file (eeg_microstates.py). The record contains 192 s. from an eyes-closed resting state experiment of a healthy male subject recorded with a 30 channel EEG cap in the standard 10–10 electrode configuration. The experiment was approved by the local ethics committee of the Goethe University, Frankfurt, Germany. The EEG sampling rate is 250 Hz and the data is band-pass filtered to the 1–40 Hz range. Electrode locations are given as cartesian coordinates in the cap.xyz file which is imported for visualization of EEG topographies. All files are contained in our GitHub repository. To import EEG data, the package contains a basic edf file reader, using the publicly available specifications for the .edf file format. Data is loaded in a single line of code:

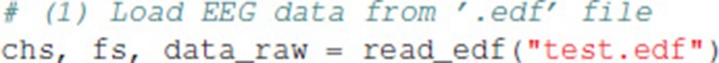



The first two return variables are a list of channel name strings and the sampling frequency in Hz. EEG data is contained in a NumPy array of shape (n_t_, n_ch_), with time samples along the rows (first index) and electrodes or channels along columns (second index). In case the provided functions are called from another Python program, EEG data must be formatted into that shape to be processed by our functions.

EEG data is usually pre-processed by a band-pass filter. As an example, we give the code for a pass band of 1–35 Hz, where fs denotes the sampling frequency in Hz and the data array has the time axis running along the first dimension (axis = 0):

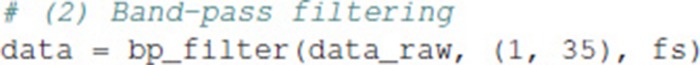



**Figure 2:**
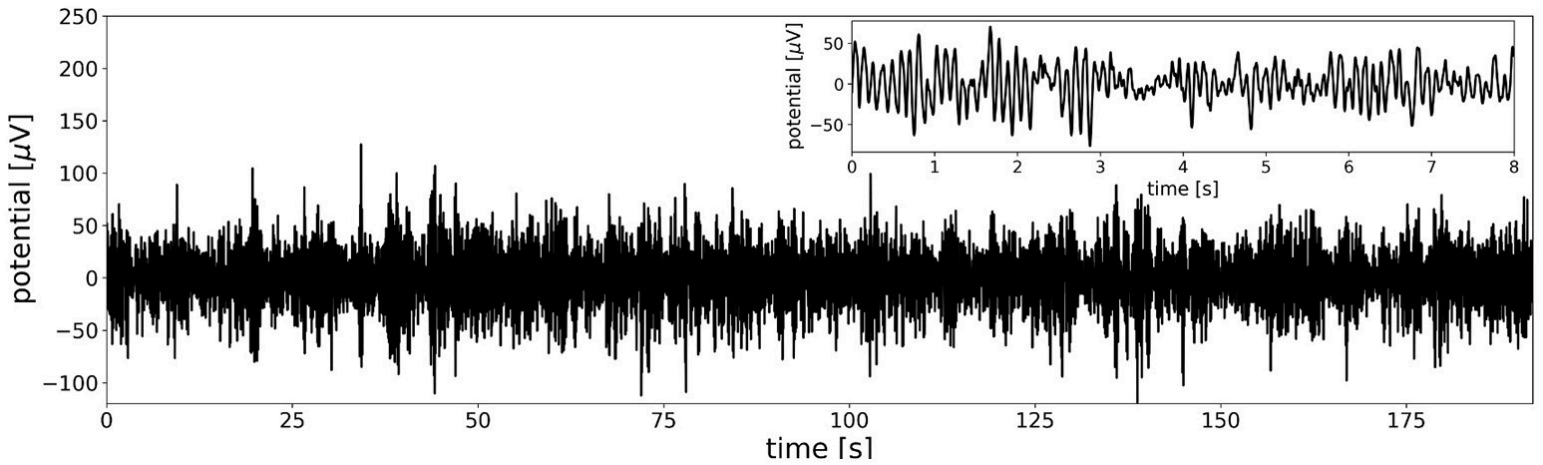
The overall structure of the EEG time series is visualized by the first principal component of the data set. The first principal component of the test file yields a time series similar to the signals observed at single electrodes, especially at parietal and occipital locations. The main feature of the data set are amplitude modulated oscillations in the alpha frequency band (8–12 Hz). The inset in the upper right corner shows the first 8 s of data to better visualize the characteristic alpha oscillations at a shorter time scale.

To get a general impression of the data in one dimension, an option to plot the time-course of the first principal component of the multi-channel data set is included. Figure 2 shows that the time series contains strongly amplitude modulated, irregular oscillations in the alpha frequency band. The inset in the upper right corner shows the first 8 s. of the data to illustrate alpha oscillations on a shorter time scale, as often found in EEG visualization software.

### 3.2. Modified K-means clustering and competitive fitting

Microstates are computed by the modified K-means clustering algorithm introduced in Pascual-Marqui et al. (1995) and as reviewed in Murray et al. (2008). The algorithm receives the EEG data array (n_t_ × n_ch_) and the desired number of microstate maps n_maps_ as minimum inputs. Note that the number of microstates is an optional argument that can be set to any integer n_maps_ ≥ 2. If not provided, the default value n_maps_ = 4 is used. In the worked example, four microstates are computed. The remaining parameters (n_runs_, maxerr, maxiter) can be provided as additional parameters to the kmeans function call, otherwise the default values (n_runs_ = 10, maxerr = 10^−6^, maxiter = 500) are used. From all K-means runs, the optimum run is selected according to the cross-validation (CV) criterion detailed in Murray et al. (2008). The CV criterion to be minimized measures the residual variance while correcting for the number of electrodes. Note that Murray recommends to (spatially) downsample EEG data with more than 64 electrodes (Murray et al., 2008). The function call looks like:

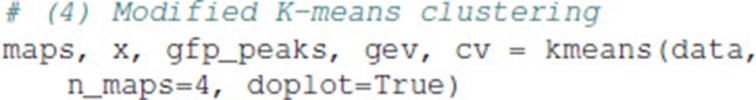



The microstate maps are returned in a (n_maps_, n_ch_) formatted array, and the variable *x* contains the sequence of microstate map labels for the given EEG data series, i.e., its length is equal to the number of EEG time samples. The remaining return values contain the indices of the GFP peaks used for clustering, the global explained variance (GEV) of the microstate maps, and the absolute value of the cross-validation criterion, i.e., the minimum value across all K-means runs.

The microstate ordering returned by the K-means algorithm is random, whereas a standard ordering has been established in the literature (Koenig et al., 2002). Our K-means implementation contains an option to re-label the microstates interactively before proceeding. In the case of four microstates, the standard microstate labeling is based on the map geometry. For map A, the border between positive and negative potential values runs approximately along the diagonal from the frontal left to the occipital right corners. Map B is diagonal in the opposite direction, map C has a horizontal orientation, and map D is often circular. Sometimes, slightly different maps are generated. We mostly observed the occurrence of a map D with a vertical axis instead of a circular pattern. In this case, you can either accept the results and proceed, or re-cluster the data set as the results of the K-means algorithm differ between runs.

Note that the sequence returned by the K-means algorithm contains the microstate labels as numbers, {*A, B, C, D*} → {0, 1, 2, 3}, in order to accelerate information-theoretical computations. Using numbers, the microstate label can directly be used as an index in arrays and matrices. If microstate sequences generated by external software are analyzed, the labels have to be converted to the numerical values 0, …, n_maps_ − 1 before using the functions of this package.

### 3.3. Information-theoretical analysis-motivation and basics

To analyze the microstate sequence with information-theoretical methods, we first need to compute the distribution of microstate labels *P*(*X*_*t*_ = *S*_*i*_), i.e., the probability to find the label *S*_*i*_ ∈ {*A, B, C, D*} at time point *t*. The distribution of *X*_*t*_ can be characterized by its Shannon entropy *H* (Kullback et al., 1962):

(1) $$\begin{array}{rc} H & =-\underset{i}{\sum }P\left(X_{t}=S_{i}\right)\log P\left(X_{t}=S_{i}\right). \end{array}$$

If the sequence always repeated the same microstate label, uncertainty would be minimal and its Shannon entropy would attain its minimum value *H* = 0, corresponding to a delta distribution *P*(*X*_*t*_). Maximum entropy is obtained for a uniform distribution of microstate labels, resulting in *H* = log(4) in the case of four microstates. Logarithms are taken with respect to the base *e* (Euler's constant), leading to the unit “nats.”

The subsequent tests will take into account dependencies between microstate labels at different times. For one-step transitions *X*_*t*_ → *X*_*t*+1_, dependencies on the values *X*_*t*_, *X*_*t*−1_, and *X*_*t*−2_ are tested by the Markovianity tests, as detailed below. For further time lags, *X*_*t*+*k*_ with *k* > 1, temporal dependencies are assessed by mutual information between *X*_*t*_ and *X*_*t*+*k*_. Moreover, we test time stationarity and the symmetry of the transition matrix. Each test leads to specific distributions, an empirical distribution derived from the actual data, and a reference distribution derived from the independence assumption under the null hypothesis. The distance between the empirical distribution (*p*_*i*_) and the null distribution (*q*_*i*_) is measured by the Kullback-Leibler divergence $D\left(p,q\right)=-\underset{i}{\sum }p_{i}\log\frac{p_{i}}{q_{i}}$ (Kullback, 1959; Kullback et al., 1962). Statistical significance is tested with χ^2^-statistics using classical convergence theorems (Anderson and Goodman, 1957; Kullback, 1959; Billingsley, 1961; Kullback et al., 1962).

The specific test statistics along with their mathematical expressions are given in Table 1. In notation, we follow (Kullback et al., 1962) and denote observed frequencies by *f*. The estimated probability of microstate label *S*_*i*_, denoted as *p*_*i*_, is the ratio of *f*_*i*_, the number of observations of label *S*_*i*_, and the sample size *n*, or $p_{i}=\frac{f_{i}}{n}$. In Table 1, indices run over microstate labels and multiple sums are abbreviated by a single summation sign and the indices over which the sum is calculated.

**Table 1:** Test statistics for microstate sequences.

**Property**	**Expression**	**G-test statistic**	**d.o.f**.
Markov-0	*P*(*X*_*t*+1_ | *X*_*t*_) = *P*(*X*_*t*+1_)	$G_{0}=2\underset{ij}{\sum }f_{ij}\log\frac{nf_{ij}}{f_{i}f_{j}}$	(*n*_*s*_ − 1)(*n*_*s*_ − 1)
Markov-1	*P*(*X*_*t*+1_ | *X*_*t*_, *X*_*t*−1_) = *P*(*X*_*t*+1_ | *X*_*t*_)	$G_{1a}=2\underset{ijk}{\sum }f_{ijk}\log\frac{f_{ijk}f_{j}}{f_{ij}f_{jk}}$	*n*_*s*_(*n*_*s*_ − 1)(*n*_*s*_ − 1)
Geometric	$q_{i}\left(k\right)=\left(1-T_{ii}\right)\times T_{ii}^{k-1}$	$G_{1b}=2\overset{m}{\underset{i=1}{\sum }}p_{i}\log\frac{p_{i}}{q_{i}}$	*m*−1
Markov-2	*P*(*X*_*t*+1_ | *X*_*t*_, *X*_*t*−1_, *X*_*t*−2_) = *P*(*X*_*t*+1_ | *X*_*t*_, *X*_*t*−1_)	$G_{2}=2\underset{ijkl}{\sum }f_{ijkl}\log\frac{f_{ijkl}f_{jk}}{f_{ijk}f_{jkl}}$	*n*_*s*_*n*_*s*_(*n*_*s*_ − 1)(*n*_*s*_ − 1)
Stationarity	$P\left(X_{t+1}^{\left(k\right)}|X_{t}^{\left(k\right)}\right)=P\left(X_{t+1}|X_{t}\right)$	$G_{3}=2\underset{ijk}{\sum }f_{ijk}\log\frac{f_{ijk}f_{j}}{f_{ij}f_{jk}}$	(*r*−1)(*n*_*s*_ − 1)*n*_*s*_
Symmetry	*P*(*X*_*t*+1_ = *S*_*i*_ | *X*_*t*_ = *S*_*j*_) = *P*(*X*_*t*+1_ = *S*_*j*_ | *X*_*t*_ = *S*_*i*_)	$G_{4}=2\underset{i\neq j}{\sum }f_{ij}\log\frac{2f_{ij}}{f_{ij}+f_{ji}}$	*n*_*s*_(*n*_*s*_ − 1)/2

### 3.4. Symbol distribution and the transition matrix

Basic statistics commonly used to characterize microstate sequences can be obtained by calling:

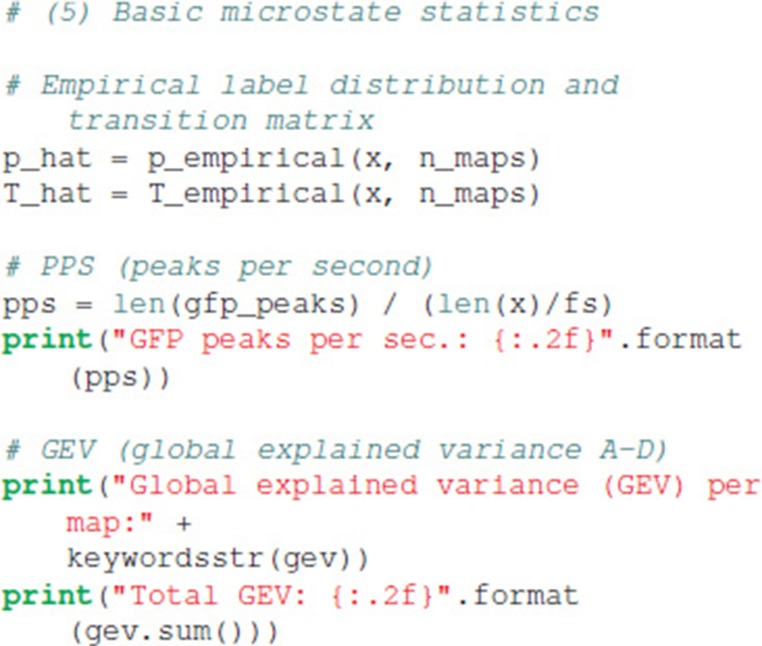



The tutorial code outputs the basic statistics on the console. The distribution of the four microstate maps is *p* = [0.279, 0.222, 0.235, 0.264], identical to the sometimes used term “ratio of time covered” (RTT) (Brodbeck et al., 2012). The first order transition matrix *T* evaluates to:

$$T_{\text{sym}}=\left(\begin{array}{cccc} 0.799 & 0.071 & 0.069 & 0.061\\ 0.079 & 0.771 & 0.094 & 0.057\\ 0.053 & 0.064 & 0.767 & 0.116\\ 0.099 & 0.061 & 0.056 & 0.784 \end{array}\right)$$

The EEG data set at hand contains 21.51 GFP peaks per second, and the global explained variance (GEV) per microstate map in our case was 0.13 (map A), 0.10 (map B), 0.31 (map C), and 0.19 (map D), giving the total GEV of this run equal to 0.73. Due to the stochastic initialization of the K-means algorithm, other runs of the algorithm may give slightly different GEV values.

The Shannon entropy of the sequence and the maximum entropy possible for the sequence are computed as:

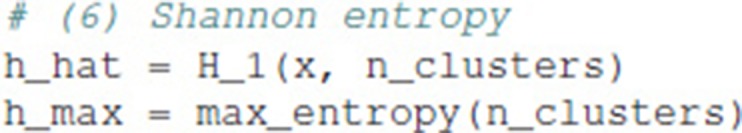



The empirical Shannon entropy of the microstate sequence is *H* = 1.38, while the maximum possible Shannon entropy for any series of four symbols is log(4) = 1.39. We see that the EEG derived sequence almost achieves maximum entropy, suggesting a process with high randomness. In the following sections, however, we show how distinct memory features such as periodicities linked to the cortical alpha rhythm can be extracted and used to characterize the sequence.

### 3.5. Markov properties and markovianity tests

First, we test if the sequence follows a simple Markov process of order 0, 1, or 2. To this end, the following tests will be computed:

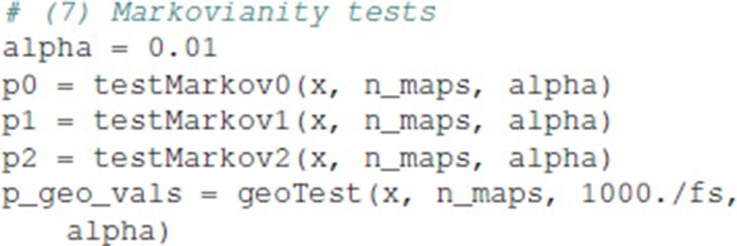



The console output for all tests also gives the value of the test statistic and the degrees of freedom of the corresponding χ^2^ distribution, calculated according to Table 1.

#### 3.5.1. Zero-order markov property

The null hypothesis is that the transition probability from the current state *X*_*t*_ to the next state *X*_*t*+1_ is independent on *X*_*t*_. Therefore, *P*(*X*_*t*+1_ | *X*_*t*_) = *P*(*X*_*t*+1_) under the null hypothesis. We obtain the corresponding test statistic *G*_0_ in Table 1, if the observed number of transitions *X*_*t*_ = *S*_*i*_ → *X*_*t*+1_ = *S*_*j*_ is denoted *f*_*ij*_, the number of observations *X*_*t*_ = *S*_*i*_ is *f*_*i*_, and the number of observations *X*_*t*+1_ = *S*_*j*_ is *f*_*j*_. The length of the microstate sequence *X*_*t*_ is *n*. The degrees of freedom (d.o.f.) of the asymptotic χ^2^ distribution is given in the right column of Table 1 (Kullback, 1959; Kullback et al., 1962). The console output shows that the zero-order Markovianity test yields a p-value of almost zero, within double floating point precision, under the assumption of total independence between subsequent symbols. The independence assumption is therefore clearly rejected.

#### 3.5.2. First-order markov property

The null hypothesis is that the transition probability *P*(*X*_*t*+1_ | *X*_*t*_) only depends on *X*_*t*_, and not on any states further in the past, implying *P*(*X*_*t*+1_ | *X*_*t*_, *X*_*t*−1_) = *P*(*X*_*t*+1_ | *X*_*t*_). Calculating the test statistic *G*_1*a*_ as given in Table 1, we find *p* = 4.29 × 10^−145^, indicating that a first-order Markov process is also rejected as a data model. Alternatively, first-order Markovianity can be assessed based on the equivalence of the first-order Markov property (the memoryless property) with a geometric distribution of state durations (Feller, 1971). Each microstate label has an associated lifetime distribution that contains the lengths of contiguous segments of the given label. For a first-order Markov process, the probability that label *i* appears in a contiguous segment of length *k* follows the geometric distribution $q_{i}\left(k\right)=\left(1-T_{ii}\right)\times T_{ii}^{k-1}$. The term $T_{ii}^{k-1}$ is the (*k* − 1)-th potency of the *i*-th diagonal element of the transition matrix *T*. In the G-test statistic *G*_1*b*_ in Table 1, *m* is the maximum lifetime and *p*_*i*_ is the empirical lifetime distribution. Testing our EEG data set for geometric lifetime distributions gives analogous results to the test statistic *G*_1*a*_, rejecting the first-order Markov hypothesis for all four microstate maps (*p*_*A*_ = 1.33 × 10^−19^, *p*_*B*_ = 2.68 × 10^−17^, *p*_*C*_ = 1.57 × 10^−75^, *p*_*D*_ = 3.24 × 10^−14^).

#### 3.5.3. Second-order markov property

Second-order Markovianity is tested based on the null hypothesis that the transition probability *P*(*X*_*t*+1_ | *X*_*t*_, *X*_*t*−1_) does not change if one step further in the past is taken into consideration. The resulting statistical expression for the null hypothesis is *P*(*X*_*t*+1_ | *X*_*t*_, *X*_*t*−1_, *X*_*t*−2_) = *P*(*X*_*t*+1_ | *X*_*t*_, *X*_*t*−1_). Note that a true first-order Markov process, as later used in surrogate data tests, should also fulfill the second-order Markov property, as neither of the states *X*_*t*−1_ or *X*_*t*−2_ contribute information about the transition probability *P*(*X*_*t*+1_ | *X*_*t*_). For the test data, second-order Markovianity is clearly rejected (*p* = 3.32 × 10^−86^).

### 3.6. Stationarity of the transition matrix

Stationarity of the transition matrix over time depends on the length *L* of the time window. For a given *L*, the data set is partitioned into *r* non-overlapping blocks of length *L* and the transition matrix is computed for each data block *k* = 0, …, *r*−1. In case of stationarity, the number of transitions *X*_*t*_ = *S*_*i*_ → *X*_*t*+1_ = *S*_*j*_ within block *k*, denoted *f*_*ijk*_, is independent of the block index *k*. The expression for the null hypothesis and the test statistic can be found in Table 1. The stationarity test is computed as:

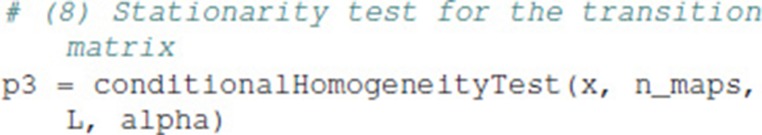



For a block size of 5,000, we obtain 9 data blocks, and the *p*-value of *p* = 2.21 × 10^−5^ indicates that the transition matrix of the test data set is not stationary. Other block sizes can be defined in the function call, or interactively in the console and IPython tutorials provided.

### 3.7. Symmetry

If each state transition occurs with the same probability as the reverse transition, the transition matrix *T* will be symmetric. The expression for the null hypothesis and the test statistic are given in Table 1, and the symmetry test is computed as:

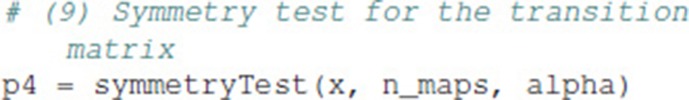



The test result *p* = 4.88 × 10^−89^ leads to rejection of the null hypothesis, and to the conclusion that the EEG data set has an asymmetric transition matrix. Asymmetry of the transition matrix is important when considering non-equilibrium processes possibly underlying microstate dynamics (von Wegner et al., 2017).

### 3.8. Markov surrogate data

**Figure 3:**
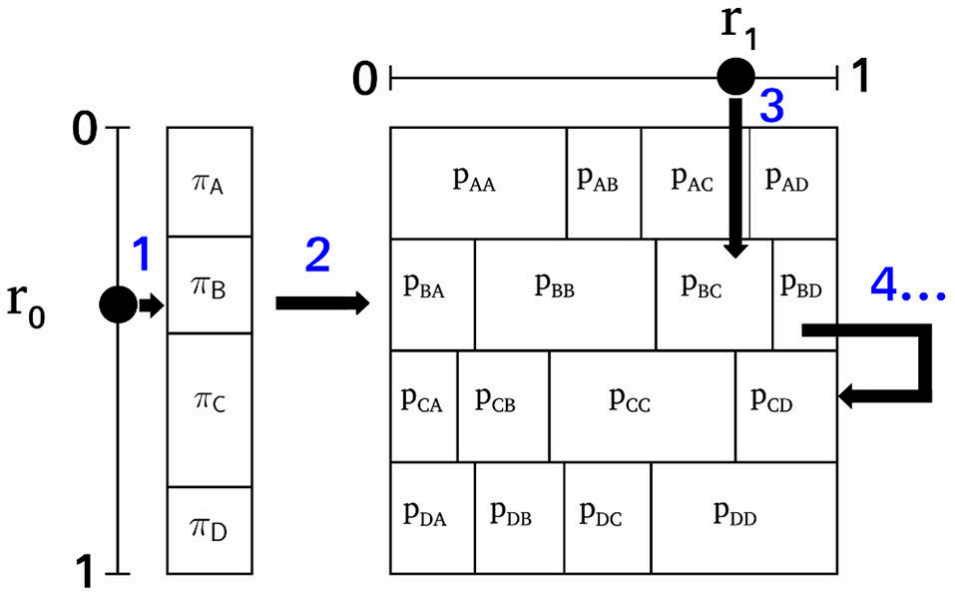
The surrogate Markov process algorithm. The initial state is selected according to the equilibrium distribution π using a pseudo-random number *r*_0_ ~ *U*[0, 1] (step 1). In this example, state *B* is selected and therefore, the next state is selected using the partition of unity defined by row 2 of the transition matrix *T* (step 2) and the pseudo-random number *r*_1_ ~ *U*[0, 1]. Here, *r*_1_ points to *p*_*BC*_ (step 3), so the next state of the process is *C*. Starting from state *C*, the successor state will be selected from the third row of *T*, and a new random number *r*_1_ (step 4). The algorithm can be iterated for any desired length of the surrogate sequence.

As a first-order Markov process is uniquely defined by an initial state distribution π and a first-order transition matrix *T*, an equivalent Markov process with π and *T* identical to the empirical microstate sequence can be synthesized (Häggström, 2002). The iterative construction is visualized in Figure 3 where the individual steps are labeled with (blue) numbers. The procedure starts with an initialization function and then iterates an updating function for the desired length of the surrogate sequence. The initial state, one of the microstate labels *A, B, C, D*, is selected in accordance with the equilibrium distribution π. A pseudo-random number *r*_0_ ~ *U*[0, 1], uniformly and independently distributed on the unit interval, defines the index of the initial state by the condition $\overset{j-1}{\underset{i=0}{\sum }}\pi_{i}\leq r_{0}<\overset{j}{\underset{i=0}{\sum }}\pi_{i}$. Step 1 in Figure 3 illustrates this step showing that the equilibrium distribution π partitions the unit interval [0, 1]. Given the initial state, all subsequent states of the surrogate sequence are generated by the transition matrix *T*. The same principle as in the initialization step is used. The current state at time *t* determines the row of the transition matrix to be used for the next transition *t* → *t* + 1. In Figure 3, the random initial state is *B*, so the next state is calculated from the second row of *T* (step 2). As the conditional probabilities in each row fulfill $\underset{j}{\sum }T_{ij}=1$, each row of *T* is a partition of the unit interval. Choosing another random variable *r*_1_ ~ *U*[0, 1] (step 3), the index of the next state is determined by $\overset{j-1}{\underset{l=0}{\sum }}T_{il}\leq r_{t}<\overset{j}{\underset{l=0}{\sum }}T_{il}$. In Figure 3, *r*_1_ points to element *p*_*BC*_, and thus, we record the state transition *B* → *C* for *t* → *t*+1. The next state is generated in the same manner (step 4), this time using the third row of *T*, because the current state is now *C*. Using NumPy, the two computational steps used by the algorithm can be written as a single line of code each.

First-order Markov surrogates are computed as:

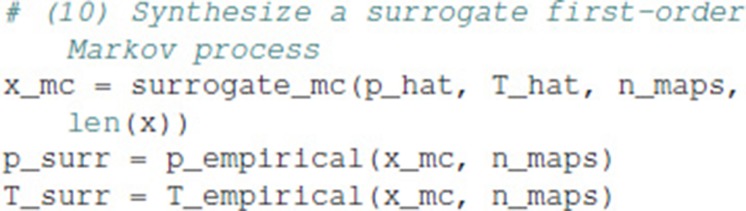



By construction, the synthetic Markov chain has a symbol distribution almost identical, within stochastic boundaries, to the one of the experimental data. Here, we get *p* = [0.281, 0.219, 0.239, 0.261] for the surrogate Markov chain. The surrogate process also has a transition matrix almost identical to the experimental data:

$$T_{\text{sym}}=\left(\begin{array}{cccc} 0.797 & 0.070 & 0.072 & 0.061\\ 0.081 & 0.769 & 0.091 & 0.059\\ 0.050 & 0.064 & 0.768 & 0.119\\ 0.105 & 0.060 & 0.059 & 0.777 \end{array}\right)$$

All tests calculated for the experimental data set can now be applied to the surrogate sequence, which by construction is a first-order Markov sequence:

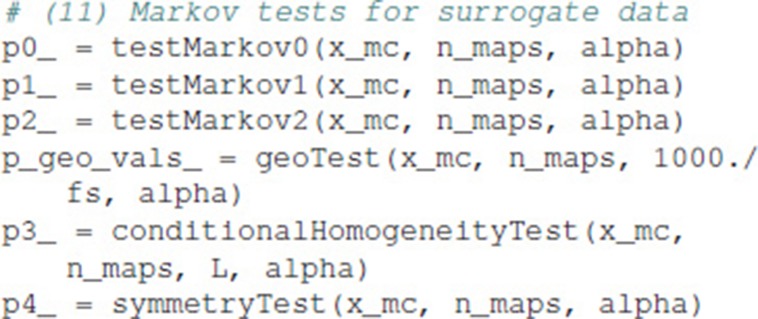



The test results reveal that all desired properties are fulfilled by the surrogate Markov sequence. The sequence is not zero-order Markov (*p* = 0.00), but is first-order Markov (*p* = 0.553) and also second-order Markov (*p* = 0.886), as expected. The alternative test for first-order Markovianity based on geometric lifetime distributions confirms the above results, as the null hypothesis is accepted for all four microstate maps (*p*_*A*_ = 0.545, *p*_*B*_ = 0.302, *p*_*C*_ = 0.207, *p*_*D*_ = 0.797). As the surrogate sequence is synthesized from a constant transition matrix, we find the sequence to be stationary (*p* = 0.180, block size 5,000) while reproducing the asymmetry of the experimental transition matrix (*p* = 3.84 × 10^−95^).

### 3.9. Mutual information

Time-lagged mutual information for discrete time lag *k* can be defined in entropy terms as:

(2) $$\begin{array}{rc} I\left(k\right) & =H\left(X_{t+k}\right)-H\left(X_{t+k}|X_{t}\right). \end{array}$$

In words, *I*(*k*) measures dependencies between time points *t* and *t*+*k* as the difference between two entropies. The term *H*(*X*_*t*+*k*_) is the uncertainty about *X*_*t*+*k*_ without further knowledge about the past, and *H*(*X*_*t*+*k*_ | *X*_*t*_) is the conditional uncertainty about *X*_*t*+*k*_, knowing the state *X*_*t*_. The time-lagged mutual information of a first-order Markov process can be written in terms of its equilibrium distribution π and its transition matrix *T* (von Wegner et al., 2017):

(3) $$\begin{array}{rc} Î\left(k\right) & =-\underset{i}{\sum }\pi_{i}\log\pi_{i}+\underset{i}{\sum }\pi_{i}\underset{j}{\sum }T_{ij}^{k}\log T_{ij}^{k}. \end{array}$$

The equation uses the matrix potency *T*^*k*^, computed using a diagonalization of *T* (von Wegner et al., 2017). The time-lagged mutual information function for the EEG microstate sequence and the Markov surrogate are calculated as:

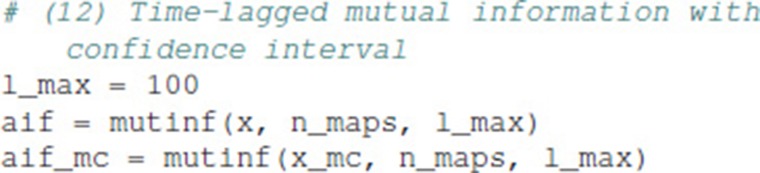



In the tutorials, a confidence interval for the mutual information function, or autoinformation function (AIF) is computed from 10 Markov surrogates, in order to limit the computation time. The results are visualized at the bottom of Figure 1. The EEG-derived autoinformation function [label AIF (EEG), solid black line and squares] lies within the α = 0.01 confidence interval defined by *n* = 10 Markov surrogate processes [label AIF (Markov), gray area]. Time lags up to 400 ms are shown on a y-semi-logarithmic scale.

The demonstration of non-Markovianity, non-stationarity and periodic information in resting state EEG recordings were the main results presented in von Wegner et al. (2017). This tutorial should enable the reader to reproduce these results with their own data, and to design new studies to further elucidate the functional role of EEG microstates.

## 4. IPython tutorial

We provide an interactive IPython notebook to illustrate a typical analysis pipeline starting from raw EEG and leading to test statistics and graphical presentations of the results. The analysis proceeds in the same order as the console tutorial, and using identical numbering of all steps for easier comparison. The notebook is part of the repository and can be viusalized on the GitHub page directly, or using the nbviewer web application, pasting the eeg_microstates package url. The only exception is the iframe element calling the PubMed site for reviewing recent publications on the topic, which is not rendered on these pages for security reasons. In a running notebook, however, the link is rendered interactively.

### 4.1. Acceleration

The published code can be further optimized with respect to computational speed. As several functions involve nested loops over simple numerical values, well known Python accelerators can be applied. We tested Numba just-in-time compilation, compiled C functions using Cython, pure C functions invoked by a system call from Python and external Julia code. All methods gave considerable speedups which we do not further quantify here. We anticipate users to choose their favorite method. Though we did not include these code variants in the published package, in order to minimize dependencies and to maximize portability of the code across platforms, additional code can be obtained from the authors.

## 5. Discussion and outlook

In the present article, we introduce an open-source Python package to perform the microstate algorithm on EEG data sets, and to analyze the resulting symbolic time series using information-theoretic measures and statistical tests. We presented the application of the procedures included in the package in a recent publication (von Wegner et al., 2017). As the methods we used in the paper are not available as open-source code, to the best of our knowledge, the code is presented alongside with the theoretical basis and a tutorial. We focused on code portability in order to provide easy access of the algorithms presented here. Useful applications of the package include the comparison of information-theoretical quantities under varying experimental conditions. For instance, in the past we have used the transition matrix approach to quantify microstate sequences calculated from EEG recordings during wakefulness and non-REM sleep in healthy subjects and in synaesthesia patients (Brodbeck et al., 2012; Kuhn et al., 2015). Using the new algorithms, we can extend these analyses and add spectral information, in particular the peaks of the time-lagged mutual information function, to search for subtle differences in the temporal structure of microstate sequences. Also, the (non-)stationarity of microstate sequences can be compared under different conditions, and prior to using other algorithms requiring stationarity. The same principle can be followed to study EEG recordings (resting state or ERPs) in neuropsychiatric diseases or during cognition.

## Author contributions

FvW implemented the code, performed software tests and wrote the manuscript and tutorials. HL provided EEG data and co-designed the analysis pipeline and software structure.

## Conflict of interest statement

The authors declare that the research was conducted in the absence of any commercial or financial relationships that could be construed as a potential conflict of interest.

## References

[B1] Anderson T. W. Goodman L. A. (1957). Statistical inference about Markov chains. Ann. Math. Stat. 28, 89–110.

[B2] Antonova I. Bänninger A. Dierks T. Griskova-Bulanova I. Koenig T. Kohler A. (2015). Differential recruitment of brain networks during visuospatial and color processing: evidence from ERP microstates. Neuroscience 305, 128–138. 10.1016/j.neuroscience.2015.07.078 26241335

[B3] Billingsley P. (1961). Statistical methods in Markov chains. Annals Math. Stat. 32, 12–40.

[B4] Brodbeck V. Kuhn A. von Wegner F. Morzelewski A. Tagliazucchi E. Borisov S.. (2012). EEG microstates of wakefulness and NREM sleep. Neuroimage 62, 2129–2139. 10.1016/j.neuroimage.2012.05.060 22658975

[B5] Brunet D. Murray M. M. Michel C. M. (2011). Spatiotemporal analysis of multichannel EEG: CARTOOL. Comput. Intell. Neurosci. 2011:813870. 10.1155/2011/813870 21253358 PMC3022183

[B6] Dinov M. Leech R. (2017). Modeling uncertainties in eeg microstates: analysis of real and imagined motor movements using probabilistic clustering-driven training of probabilistic neural networks. Front. Hum. Neurosci. 11:534. 10.3389/fnhum.2017.00534 29163110 PMC5671986

[B7] Feller W. (1971). An Introduction to Probability Theory and Its Applications, Vol. II, 2nd Edn. New York, NY: John Wiley & Sons Inc.

[B8] Häggström O. (2002). Finite Markov Chains and Algorithmic Applications. Cambridge: Cambridge University Press.

[B9] Iannilli E. Broy F. Kunz S. Hummel T. (2017). Age-related changes of gustatory function depend on alteration of neuronal circuits. J. Neurosci. Res. 95, 1927–1936. 10.1002/jnr.24071 28493338

[B10] Iannilli E. Wiens S. Arshamian A. Seo H.-S. (2013). A spatiotemporal comparison between olfactory and trigeminal event-related potentials. Neuroimage 77, 254–261. 10.1016/j.neuroimage.2012.12.057 23298751

[B11] Khanna A. Pascual-Leone A. Michel C. M. Farzan F. (2015). Microstates in resting-state EEG: current status and future directions. Neurosci. Biobehav. Rev. 49, 105–113. 10.1016/j.neubiorev.2014.12.010 25526823 PMC4305485

[B12] Koenig T. Lehmann D. Merlo M. C. Kochi K. Hell D. Koukkou M. (1999). A deviant EEG brain microstate in acute, neuroleptic-naive schizophrenics at rest. Eur. Arch. Psychiatry Clin. Neurosci. 249, 205–211. 10449596 10.1007/s004060050088

[B13] Koenig T. Prichep L. Lehmann D. Sosa P. V. Braeker E. Kleinlogel H.. (2002). Millisecond by millisecond, year by year: normative EEG microstates and developmental stages. Neuroimage 16, 41–48. 10.1006/nimg.2002.1070 11969316

[B14] Kuhn A. Brodbeck V. Tagliazucchi E. Morzelewski A. von Wegner F. Laufs H. (2015). Narcoleptic patients show fragmented EEG-microstructure during early NREM sleep. Brain Topogr. 28, 619–635. 10.1007/s10548-014-0387-1 25168255

[B15] Kullback S. (1959). Information Theory and Statistics. Mineola, NY: Dover Publications, Inc.

[B16] Kullback S. Kupperman M. Ku H. H. (1962). Tests for contingency tables and Markov chains. Technometrics 4, 573–608.

[B17] Lehmann D. Faber P. L. Galderisi S. Herrmann W. M. Kinoshita T. Koukkou M.. (2005). EEG microstate duration and syntax in acute, medication-naive, first-episode schizophrenia: a multi-center study. Psychiatry Res. 138, 141–156. 10.1016/j.pscychresns.2004.05.007 15766637

[B18] Lehmann D. Ozaki H. Pal I. (1987). EEG alpha map series: brain micro-states by space-oriented adaptive segmentation. Electroencephalogr. Clin. Neurophysiol. 67, 271–288. 2441961 10.1016/0013-4694(87)90025-3

[B19] Milz P. (2016). Keypy–an open source library for EEG microstate analysis. Eur. Psychiatr. 33, S290–S643. 10.1016/j.eurpsy.2016.01.1812

[B20] Milz P. Faber P. L. Lehmann D. Koenig T. Kochi K. Pascual-Marqui R. D. (2015). The functional significance of EEG microstates-associations with modalities of thinking. Neuroimage 125, 643–656. 10.1016/j.neuroimage.2015.08.023 26285079

[B21] Murray M. M. Brunet D. Michel C. M. (2008). Topographic ERP analyses: a step-by-step tutorial review. Brain Topogr. 20, 249–264. 10.1007/s10548-008-0054-5 18347966

[B22] Niedermeyer E. da Silva F. L. (2005). Electroencephalography. Basic Principles, Clinical Applications, and Related Fields, 5th Edn. Philadelphia, PA: Lippincott Williams & Wilkins.

[B23] Nishida K. Morishima Y. Yoshimura M. Isotani T. Irisawa S. Jann K.. (2013). EEG microstates associated with salience and frontoparietal networks in frontotemporal dementia, schizophrenia and Alzheimer's disease. Clin. Neurophysiol. 124, 1106–1114. 10.1016/j.clinph.2013.01.005 23403263

[B24] Pascual-Marqui R. D. Michel C. M. Lehmann D. (1995). Segmentation of brain electrical activity into microstates: model estimation and validation. IEEE Trans. Biomed. Eng. 42, 658–665. 7622149 10.1109/10.391164

[B25] Rossum G. (1995). Python Reference Manual. Amsterdam: Centre for Mathematics and Computer Science.

[B26] Tagliazucchi E. von Wegner F. Morzelewski A. Brodbeck V. Laufs H. (2012). Dynamic BOLD functional connectivity in humans and its electrophysiological correlates. Front. Hum. Neurosci. 6:339. 10.3389/fnhum.2012.00339 23293596 PMC3531919

[B27] Van de Ville D. Britz J. Michel C. M. (2010). EEG microstate sequences in healthy humans at rest reveal scale-free dynamics. Proc. Natl. Acad. Sci. U.S.A. 107, 18179–18184. 10.1073/pnas.1007841107 20921381 PMC2964192

[B28] von Wegner F. Tagliazucchi E. Brodbeck V. Laufs H. (2016). Analytical and empirical fluctuation functions of the EEG microstate random walk - short-range vs. long-range correlations. Neuroimage 141, 442–451. 10.1016/j.neuroimage.2016.07.050 27485754

[B29] von Wegner F. Tagliazucchi E. Laufs H. (2017). Information-theoretical analysis of resting state EEG microstate sequences - non-Markovianity, non-stationarity and periodicities. Neuroimage 158, 99–111. 10.1016/j.neuroimage.2017.06.062 28673879

[B30] Wackermann J. Lehmann D. Michel C. M. Strik W. K. (1993). Adaptive segmentation of spontaneous EEG map series into spatially defined microstates. Int. J. Psychophysiol. 14, 269–283. 8340245 10.1016/0167-8760(93)90041-m

